# The discovery of potential acetylcholinesterase inhibitors: A combination of pharmacophore modeling, virtual screening, and molecular docking studies

**DOI:** 10.1186/1423-0127-18-8

**Published:** 2011-01-21

**Authors:** Shin-Hua Lu, Josephine W Wu, Hsuan-Liang Liu, Jian-Hua Zhao, Kung-Tien Liu, Chih-Kuang Chuang, Hsin-Yi Lin, Wei-Bor Tsai, Yih Ho

**Affiliations:** 1 Graduate Institute of Biotechnology, National Taipei University of Technology, 1 Sec. 3 ZhongXiao E. Rd., Taipei, 10608, Taiwan; 2 Department of Chemical Engineering and Biotechnology, National Taipei University of Technology, 1 Sec. 3 ZhongXiao E. Rd., Taipei, 10608, Taiwan; 3 Chemical Analysis Division, Institute of Nuclear Energy Research, 1000, Wunhua Rd., Longtan Township, Taoyuan County, 32546, Taiwan; 4 Division of Genetics and Metabolism, Department of Medical Research, Mackay Memorial Hospital, 92, Sec. 2, Chung-Shan N. Rd., Taipei, 10449, Taiwan; 5 College of Medicine, Fu-Jen Catholic University, 510 Chung Cheng Rd, Hsinchuang, Taipei County, 24205, Taiwan; 6 Department of Chemical Engineering, National Taiwan University, 1 Sec. 4 Roosevelt Rd., Taipei, 106, Taiwan; 7 School of Pharmacy, Taipei Medical University, 250 Wu-Hsing St., Taipei, 110, Taiwan

## Abstract

**Background:**

Alzheimer's disease (AD) is the most common cause of dementia characterized by progressive cognitive impairment in the elderly people. The most dramatic abnormalities are those of the cholinergic system. Acetylcholinesterase (AChE) plays a key role in the regulation of the cholinergic system, and hence, inhibition of AChE has emerged as one of the most promising strategies for the treatment of AD.

**Methods:**

In this study, we suggest a workflow for the identification and prioritization of potential compounds targeted against AChE. In order to elucidate the essential structural features for AChE, three-dimensional pharmacophore models were constructed using Discovery Studio 2.5.5 (DS 2.5.5) program based on a set of known AChE inhibitors.

**Results:**

The best five-features pharmacophore model, which includes one hydrogen bond donor and four hydrophobic features, was generated from a training set of 62 compounds that yielded a correlation coefficient of R = 0.851 and a high prediction of fit values for a set of 26 test molecules with a correlation of R^2 ^= 0.830. Our pharmacophore model also has a high Güner-Henry score and enrichment factor. Virtual screening performed on the NCI database obtained new inhibitors which have the potential to inhibit AChE and to protect neurons from Aβ toxicity. The hit compounds were subsequently subjected to molecular docking and evaluated by consensus scoring function, which resulted in 9 compounds with high pharmacophore fit values and predicted biological activity scores. These compounds showed interactions with important residues at the active site.

**Conclusions:**

The information gained from this study may assist in the discovery of potential AChE inhibitors that are highly selective for its dual binding sites.

## Background

Acetylcholinesterase (AChE), one of the most essential enzymes in the family of serine hydrolases, catalyzes the hydrolysis of neurotransmitter acetylcholine, which plays a key role in memory and cognition [1-3]. While the physiological role of the AChE in neural transmission has been well known, it is still the focus of pharmaceutical research, targeting in treatments of myasthenia gravis, glaucoma, and Alzheimer's disease (AD). It has been elucidated that cholinergic deficiency is associated with AD [4]; therefore, one of the major therapeutic strategies is to inhibit the biological activity of AChE, and hence, to increase the acetylcholine level in the brain. Currently, most of the drugs used for the treatment of AD are AChE inhibitors, including the synthetic compounds tacrine, donepezil, and rivastigmine, which have all been proven to improve the situation of AD patients to some extent. So far, the four drugs that have been approved by the Food and Drug Administration (FDA) to treat AD in the US are tacrine, rivastigmine (E2020), donepezil, and galanthamine, which all have some success in slowing down neurodegeneration in AD patients.

In the past decade, it has been found that AChE is involved in pathogenesis of AD through a secondary noncholinergic function associated with its peripheral anionic site. Recent findings support the enzyme's role in mediating the processing and deposition of Aβ peptide by colocalizing with Aβ peptide deposits in the brain of AD patients and promoting Aβ fibrillogenesis through the formation of stable AChE-Aβ complexes. The formation of these complexes promotes Aβ aggregation as an early event in the neurodegenerative cascade of AD [5,6] and results in cognitive impairment in doubly transgenic mice expressing human amyloid precursor protein (APP) and human AChE [7,8]. Based on these new findings, the recent design of novel classes of AChE inhibitors as therapeutic intervention for AD has been shifted toward blocking the peripheral site of AChE, the Aβ recognition zone within the enzyme [9], thereby affect the AChE-induced Aβ aggregation and thus, modulate the progression of AD.

X-ray structures of AChE co-crystallized with various ligands [10-14] provided insights into the essential structural elements and motifs central to its catalytic mechanism and mode of acetylcholine (ACh) processing. One of the striking structural features of the AChE revealed from the X-ray analysis is the presence of a narrow, long, hydrophobic gorge which is approximately 20 Å deep [15,16]. The enzyme has a catalytic triad consisting of Ser203, His447, and Glu334 [17] located in the active site of the narrow deep gorge, the lining of which contains mostly aromatic residues that form a narrow entrance to the catalytic Ser203 [16]. A peripheral anionic site (PAS) comprising another set of aromatic residues Tyr72, Tyr124, Trp286, Tyr341, and Asp74 [18] is located at the rim of the gorge and provides a binding site for allosteric modulators and inhibitors. The interaction between highly potent inhibitors, such as tacrine and donepezil, and the enzyme is characterized by cation-π interactions between the protonated nitrogens and the conserved aromatic residues, tryptophan (Trp86) and phenylalanine (Phe337). Moreover, π-π stacking between the aromatic moieties of the inhibitors and the aromatic amino acids mentioned above, as well as ion-ion-interactions between the protonated nitrogens of the inhibitors and the anionic aspartic acid (Asp72) all play crucial roles in ligand binding [15]. Most ligands, as observed from their crystal structures, are located at the bottom of the gorge that forms a wide hydrophobic pocket base, although larger ligands such as decamethonium [10] and donepezil [19] extend to the mouth of the gorge, the opening of the hydrophobic pocket.

The drug discovery process is both time-consuming and expensive [20] yet new drugs are required to satisfy the numerous unmet clinical needs in many disease indications. The number of potential target 3D structures is increasing in the Protein Data Bank (PDB) [19] and the number of drug/lead-like compounds is estimated to be at least 10^24 ^[21]. Therefore, to deal with such a large amount of data and to facilitate the drug discovery process, *in silico *virtual screening and computer-aided drug design have become increasingly important [22]. Virtual screening provides an inexpensive and fast alternative to high-throughput screening for discovering new drugs. The binding of ligand to receptor is driven in part by shape complementarity and physicochemical interactions. One of the virtual screening approaches is to develop a pharmacophore query from an inhibitor, which describes the spatial arrangement of a group of essential structural features common to a set of compounds that are critical to interacting with the receptor. The pharmacophore approach is applied in drug design and takes in consideration that molecules are active at the receptor binding site because they possess both a number of chemical features that favor the target interaction site and are geometrically complementary to it. A good pharmacophore model collects important common features of molecules distributed in the 3D space and provides a rational hypothetical conformation of the primary chemical features responsible for activity; therefore, it has become an important method and has proven extremely successful not only in demonstrating structure-activity relationships, but also in the development of new drugs [23,24].

Providing that the experimentally determined high-resolution 3D structure of the target is available, ligand-based drug design can be performed in association with molecular docking, a structure-based method, and underlying scoring functions to reproduce crystallographic ligand-binding modes. These methods can be combined to identify a number of new hit compounds with potent inhibitory activity and to understand the main interactions at the binding sites. It is believed that the concurrent use of molecular docking and consensus scoring functions could readily minimize false positive and false negative errors encountered by ligand-based (pharmacophore) virtual screening. In addition, the complementation of molecular docking and pharmacophore can produce reliable true positive and true negative results in the subsequent virtual screening procedure. The appropriate use of these methods in a drug discovery process should improve the ability to identify and optimize hits and confirm their potential to serve as scaffolds for producing new therapeutic agents.

In this study, we developed both qualitative and quantitative pharmacophore models based on AChE inhibitors collected from the same laboratory [25-33]. The pharmacophore features were used to identify potent AChE inhibitors as well as to clarify the quantitative structure-activity relationship for previously known AChE inhibitors. The best quantitative model was used as 3D search queries for screening the NCI databases to identify new inhibitors of AChE that can block both the catalytic and peripherical anionic sites. Blocking the daul-binding sites has the advantages of both preventing the degradation of acetylcholine in the brain and inhibiting the pro-aggregating effect of AChE, thus, protect neurons from Aβ toxicity. Once identified, the hit compounds were subsequently subjected to filtering by molecular docking to refine the retrieved hits. The virtual screening approach, in combination with pharmacophore modeling, molecular docking, and consensus scoring function can be used to identify and design novel AChE inhibitors with higher selectivity. The potential hit compounds obtained from this study can be further evaluated by *in vitro *and *in vivo *biological tests.

## Methods

### Data preparation

Pharmacophore modeling correlates activities with the spatial arrangement of various chemical features in a set of active analogues. The 88 AChE inhibitors in this study were collected from nine publications reported by the same laboratory [25-33], which employed similar experimental conditions and procedures to obtain bioactivity data for the compounds. The *in vitro *bioactivities of the collected inhibitors were expressed as the concentration of the test compounds that inhibited the activity of AChE by 50% (IC50). These values are generally transformed into pIC50 (-log IC50) as an expression of drug potency. Additional files 1 and 2 (Tables S1 and S2) show the structures, IC50 and pIC50 values of the inhibitors considered for this study. Among these sets, 62 diverse compounds whose binding affinities (IC50 values) ranged from 0.00106 μM to 80.5 μM (over six orders of magnitude) were selected as the training set (Additional file 1: Table S1); while the remaining 26 molecules served as the test set (Additional file 2: Table S2). The training set molecules play an important role in determining the quality of the pharmacophore models generated; while the test set compounds serve to evaluate the predictive ability of the resultant pharmacophore. Both sets of molecules must have large range of activities to obtain critical information on the pharmacophoric requirements for AChE inhibition.

The two-dimensional (2D) chemical structures of these acetylcholinesterase inhibitors (AChEIs) were sketched using CS ChemDraw Ultra (Cambridge Soft Corp., Cambridge, MA) and saved as MDL-molfile format. Subsequently, they were imported into Discovery Studio Version 2.5.5 (DS 2.5.5, Accelrys Inc., San Diego, CA) and converted into the corresponding standard three-dimensional (3D) structures. Molecular flexibility of compounds is modeled by making multiple conformers within a specific energy range. A maximum of 250 conformers for each compound were generated by the "Best quality" conformational search option based on the CHARMm force field [34], with an energy threshold of 20 kcal/mol from the lowest energy level. Default settings were kept for the other parameters.

### Pharmacophore model generation

Two different methods were applied for the ligand based pharmacophore model: HipHop and HypoGen. HipHop is generated based on the common features present in the training set molecules. HypoGen [35], an algorithm that uses the activity values of the small compounds in the training set to generate the hypothesis, was applied in this study to build the 3D QSAR pharmacophore models using DS V2.5.5 software. An automated 3D QSAR pharmacophore was created by using the activity values of compounds in the training set that includes at least 16 molecules with bioactivities spanning at least over four orders of magnitude. The wide range of bioactivities in the training set allowed for the screening of large database. The DS Feature Mapping module computed all possible pharmacophore feature mappings for the selected chemical features of the training set molecules. A minimum of 0 to a maximum of 5 features including hydrogen-bond acceptor (HBA), hydrogen-bond donor (HBD), hydrophobic (HBic), and ring aromatic (RingArom) features were selected in generating the quantitative pharmacophore model. A value of 3 was employed as the uncertainty value, which means that the biological activity of a particular inhibitor is assumed to be located somewhere in the range three times higher to three times lower of the true value of that inhibitor [35-38]. Ten pharmacophore models with significant statistical parameters were generated. The best model was selected on the basis of a highest correlation coefficient (R), lowest total cost and root mean square deviation (rmsd) values (for more details on cost values, see Ref. [39]). From the pharmacophore models generated, the relationship between the structures of the training set compounds and their experimentally determined inhibitory activities against AChE was investigated.

### Validation of the pharamacophore model

The pharmacophore models selected by correlation coefficient and cost analysis were then validated in three subsequent steps: Fischer's randomization test, test set prediction, and Güner-Henry (GH) scoring method [40-42]. First, cross validation was performed by randomizing the data using the Fischer's randomization test. Then, a test set of 26 diverse compounds with AChE inhibitory activity was selected to validate the best pharmacophore model. The test set covers similar structural diversity as the training set in order to establish the broadness of the pharmacophore predictability. All queries were performed using the Ligand Pharmacophore Mapping protocol. The GH scoring method was used following test set validation to assess the quality of the pharmacophore models. The GH score has been successfully applied to quantify model selectivity precision of hits and the recall of actives from a 3,606 molecule dataset consisting of known actives and in-actives. Of these molecules, 66 structurally and pharmacologically diverse compounds are known inhibitors of AChE that were selected from four publications [43-46]. While the other 3,540 molecules were from the previously published directory of useful decoys (DUD) dataset [47]. The DUD database, which is available for public use, was generated based on the observation that physical characteristics of the decoy background can be used for the classification of different compounds. DUD was downloaded from http://dud.docking.org (accessed July 17, 2010).

The GH scoring method was applied to the previously mentioned 66 known inhibitors of AChE and the DUD dataset molecules to validate the pharmacophore models. The method consists of computing the following: the percent yield of actives in a database (%Y, recall), the percent ratio of actives in the hit list (%A, precision), the enrichment factor *E*, and the GH score. The GH score ranges from 0 to 1, where a value of 1 signifies the ideal model.

The following is the proposed metrics for analyzing hit lists by a pharmacophore model-based database search [40-42]:

$$\begin{array}{c} \%\text{A}=\frac{\text{Ha}}{\text{A}}\times 100\\ \%\text{Y}=\frac{\text{Ha}}{\text{Ht}}\times 100\\ \text{E}=\frac{\text{Ha/Ht}}{\text{A/D}}\\ \text{GH}=\left(\frac{\text{Ha}(3\text{A}+\text{Ht})}{4\text{HtA}}\right)\left(1-\frac{\text{Ht}-\text{Ha}}{\text{D}-\text{A}}\right) \end{array}$$

%A is the percentage of known active compounds retrieved from the database (precision); Ha, the number of actives in the hit list (true positives); A, the number of active compounds in the database; %Y, the percentage of known actives in the hit list (recall); Ht, the number of hits retrieved; D, the number of compounds in the database; E, the enrichment of the concentration of actives by the model relative to random screening without any pharmacophoric approach and GH is the Güner-Henry score.

### Virtual screening

Virtual screening, an *in silico *tool for drug discovery, has been widely used for lead identification in drug discovery programs. Virtual screening methods are generally divided into ligand-based virtual screening and structure-based virtual screening. Pharmacophore-based database searching is considered a type of ligand-based virtual screening, which can be efficiently used to find novel, potential leads for further development from a virtual database. A well-validated pharmacophore model includes the chemical functionalities responsible for bioactivities of potential drugs, therefore, it can be used to perform a database search by serving as a 3D query. The best pharmacophore Hypo1 was used as a 3D structural query for retrieving potent molecules from the NCI chemical database. For each molecule in the database, the fast conformer generation method produced 250 conformers with a maximum energy tolerance of 20 kcal/mol above that of the most stable conformation.

The compounds were first filtered by Lipinski's "Rule of five" that sets the criteria for drug-like properties. Drug likeness is a property that is most often used to characterize novel lead compounds [48] by screening of structural libraries. According to this rule, poor absorption is expected if MW > 500, log P > 5, hydrogen bond donors > 5, and hydrogen bond acceptors > 10 [49]. Secondly, a molecule that satisfied all the features of the pharmacophore model used as the 3D query in database searching was retained as a hit. Two database searching options such as Fast/Flexible and Best/Flexible search are available in DS V2.5.5. Of these two, the "Best/Flexible search" yielded better results during database screening, therefore, we performed all database searching experiments using the "Best/Flexible search" option. Setting the "Maximum Omitted Features" option to zero, the best pharmacophore model was used to screen the databases for those compounds that fit all five features of the pharmacophore Hypo1. The calculations of fit values were based on how well the chemical substructures match the location constraints of the pharmacophoric features and their distance deviation from the feature centers. High fit values indicate good matches. The maximum fit value was set based on the fit value of the original ligands used to create the pharmacophore models. Those hit compounds that passed all of the screening tests were taken for further molecular docking study.

### Molecular docking

The DOCK protocols used in this study were the procedures described in our laboratory, and the methodology for their preparation has been previously studied (unpublished results). Crystal structure of AChE (PDB code: 1B41) [50], downloaded from the protein databank (PDB) [19], was used for the study. The solvent molecules were removed and hydrogen atoms were added to the protein using DS V2.5.5. Structure-based docking of 88 minimized AChE inhibitors and hits/leads from virtual screening to the active site of AChE was carried out using the LibDock program [51], which is an extension of the software DS V2.5.5. The active site was defined as the region of AChE that comes within 12 Å from the geometric centroid of the ligand. Default settings for small molecule-protein docking were used throughout the simulations. Top 50 poses were collected for each molecule with the best docked score value associated with a favorable binding conformation compared to the co-crystallized inhibitor being considered as having biological activity.

## Results

### Construction of pharmacophore model

Before the start of pharmacophore modeling, we collected a total of 88 AChE inhibitors from different literature resources. Of these compounds, 62 were carefully chosen to form a training set based on wide coverage of activity range and structural diversity. Structures and biological activities of the training set compounds are shown in Additional file 1: Table S1. The remaining compounds were included in the test set (see Additional file 2: Table S2). The top ten hypotheses were composed of HBA, HBD, HBic, and RingArom features. The values of the ten hypotheses such as pharmacophore features, root-mean-square deviations (rmsd), correlation (r), cost values, and Fischer confidence levels showed statistical significance (Table 1).

**Table 1:** The performance of 10 pharmacophoric hypotheses generated by HypoGen for AChE inhibitors

Hypotheses^a^	Pharmacophoric features in generated hypotheses	RMS deviation	Cost Values				Residual cost^d^
			**Training set (R)**^**b**^	**Error**	**Weight**	**Total**^**c**^	

1	HBD, 4×HBic	1.411	0.851	270.24	1.170	289.972	142.57
2	HBD, 4×HBic	1.416	0.850	270.73	1.338	290.628	141.91
3	HBD, 4×HBic	1.419	0.849	270.97	1.144	290.681	141.86
4	HBD, 4×HBic	1.445	0.843	273.31	1.419	293.293	139.25
5	HBD, 3×HBic, RingArom	1.469	0.836	275.44	1.243	295.242	137.30
6	HBD, 4×HBic	1.474	0.834	275.93	1.145	295.636	136.91
7	HBD, 4×HBic	1.484	0.834	276.83	1.219	296.614	135.93
8	HBD, 4×HBic	1.510	0.827	279.20	1.163	298.925	133.62
9	HBD, 4×HBic	1.514	0.826	279.61	1.262	299.432	133.11
10	HBD, 4×HBic	1.520	0.825	280.21	1.127	299.899	132.64

^a ^Fischer randomization set at 95% confidence level was performed on all pharmacophore models.

^b ^Correlation coefficient (R) between the experimental activity and the estimated fit values of the training compounds.

^c ^Total costs = error cost + weight cost + configuration cost, where configuration cost = 18.564.

^d ^Residual cost = null cost - total cost, where null cost = 432.542.

**Figure 1:**
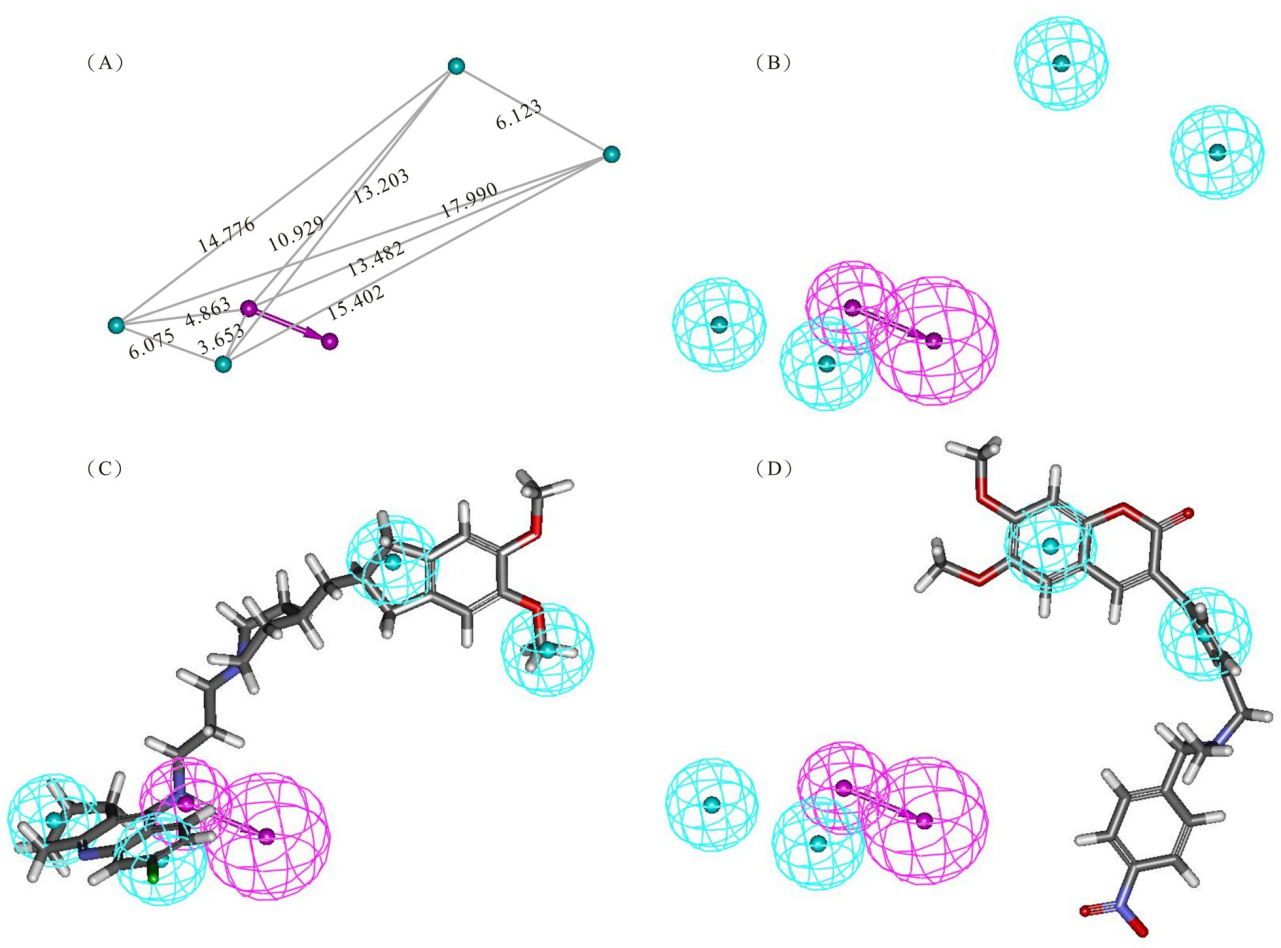
**The best Pharmacophore model (Hypo1) of AChE inhibitors generated by the HypoGen module**. (A) Three dimensional (3D) spatial arrangement and geometric parameters of Hypo1 and distance between pharmacophore features (Å). (B) Best Pharmacophore features model. (C) Hypo1 mapping with one of the most active compound 7. (D) Hypo1 mapping with one of the least active compound 44. Pharmacophore features are color-coded with light-blue for hydrophobic feature and magenta for hydrogen-bond donor.

The best hypothesis Hypo1, as shown in Figure 1, is characterized by the lowest total cost value (289.972), the highest cost difference (142.57), the lowest RMSD (1.411), and the best correlation coefficient (R = 0.851). The fixed cost and null cost are 228.233 and 432.542 bits, respectively. The total cost is low and close to the fixed cost, as well as being less and differs greatly from the null cost. All of these evidence indicate that the model, accounting for all five pharmacophore features: one hydrogen bond donor (HBD) and four hydrophobic (HBic), has good predictive ability. Figures 1A and 1B show the 3D spatial arrangement and distance constraints of all HypoGen pharmacophore features in Hypo1. The features of Hypo1 (HBD and HBic) were mapped onto the most active compound of the training set (compound **7**) shown in Figure 1C. One of the low active compound in the training set (compound **44**) was mapped partially by the features of Hypo1 (Figure 1D). Clearly, all features in the hypothesis are mapped very well with the corresponding chemical functional groups on compound **7**, while three features (i.e. one hydrogen-bond donor and two hydrophobic features) are not mapped to any functional group on compound **44**. The results of our pharmacophore study appear to validate the Hypo1 model to some extent.

### Model validation

The pharmacophore model constructed in this study was primarily validated to check for the best model that can identify the active compounds in a virtual screening process. The three steps of validation include Fischer's randomization test method, correlation of the experimental activity and the estimated fit values of the test set, and Güner-Henry (GH) scoring method.

All hypotheses were then evaluated by cross-validation using Fischer's randomization method. Validation was done by generating 19 random spreadsheets (95% confidence level) for the training set molecules and randomly reassigning activity values to each compound. The same method was used for each hypothesis to generate the random spreadsheets. The cross-validated experiment confirmed that the hypotheses have 95% significance and the results are shown in Table 1. The high statistical significance may be attributed to the significant difference between the activities of the training set molecules.

**Figure 2:**
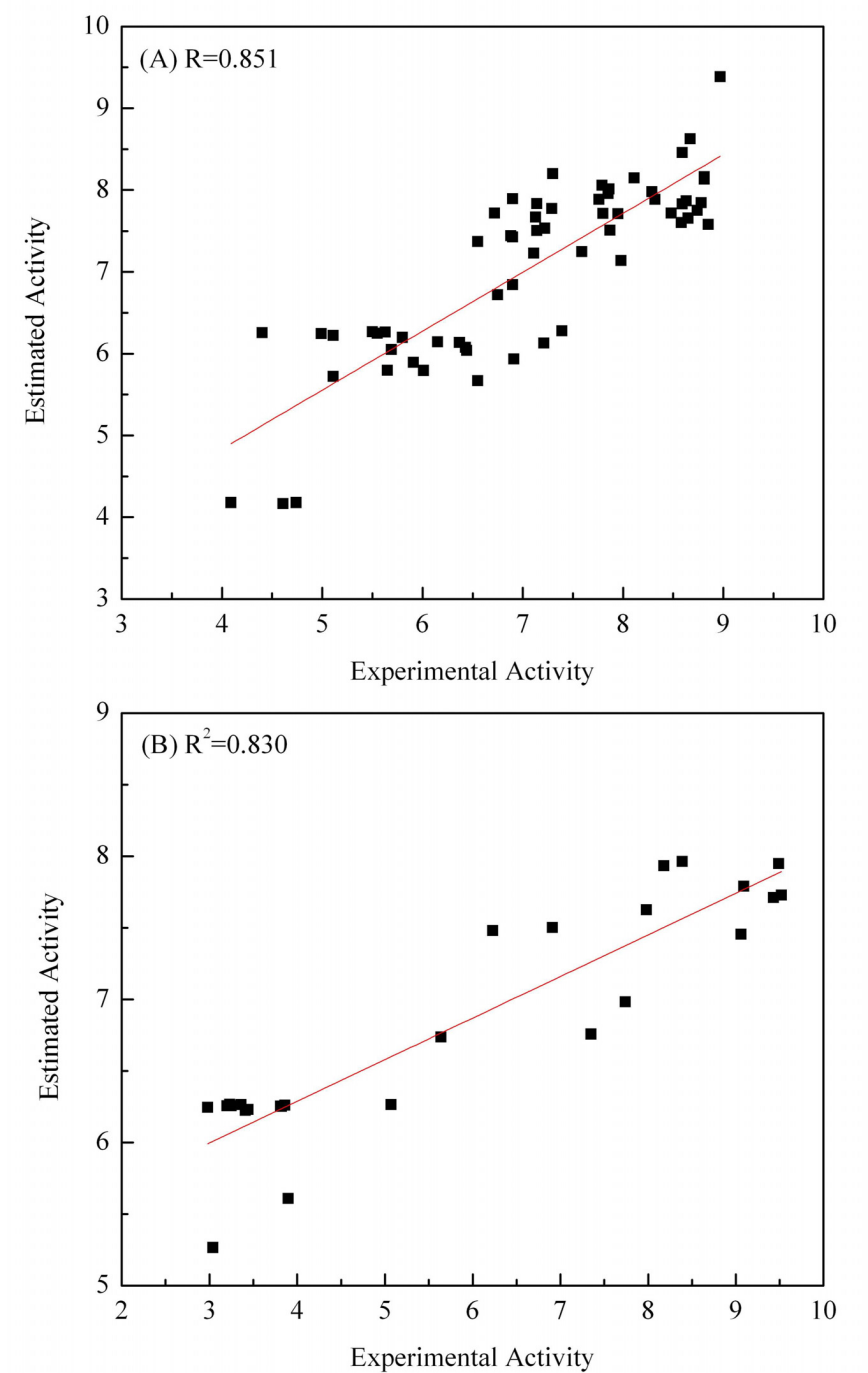
**Plot of the correlation coefficient between experimental activity and estimated fit values by Hypo 1**. (A) The training set of 62 compounds (R = 0.851) and (B) the test set of 26 compounds (R^2 ^= 0.830).

The pharmacophore model should estimate the predicted fit values of the training set molecules and accurately predict the fit values of the test set molecules. First, all ten hypotheses were evaluated using a test set of 26 known AChE inhibitors. Fit values were calculated using all ten hypotheses and correlated with experimental activities. The best hypothesis, Hypo1, showed a correlation coefficient (R^2 ^= 0.830). The correlation between the experimentally observed and estimated fit values for the training set and the test set molecules is plotted in Figure 2.

Another statistical test method used for validation includes calculation of false positives, false negatives, enrichment, and goodness of hit to determine the robustness of the generated hypotheses. Not only should the pharmacophore model generated predict the activity of the training set compounds, but it should also be capable of predicting the activities of other compounds as active or inactive. Hypo1 was used to search the known AChE inhibitors through database mining by using the BEST flexible searching technique. The results were analyzed using the hit list (Ht), number of active percent of yields (%Y), percent ratio of actives in the hit list (%A), enrichment factor (E), false negatives, false positives, and goodness of hit score (GH scoring method) (Table 2). Hypo1 succeeded in retrieving 70% of the active compounds, 22 inactive compounds (false positives), and predicted 13 active compounds as inactive (false negatives). An enrichment factor of 38.61 and a GH score of 0.73 indicated the quality of the model and high efficiency of the screening test. Overall, a strong correlation was observed between the Hypo1 predicted activity and the experimental AChE inhibitory activity (IC50) of the training and test set compounds (Figure 2). Fischer's randomization method also confirmed that the hypothesis has 95% significance, and the GH scoring method showed that the model can accurately screen for compounds with activity. These three validation procedures provided strong support for Hypo1 as the best pharmacophore model.

**Table 2:** Pharmacophore model evaluation based on the Güner-Henry scoring method

Serial No.	Parameter	AChE
1	Total molecules in database (D)	3606
2	Total Number of active in database (A)	66
3	Total Hits (Ht)	75
4	Active Hits (Ha)	53
5	% Yield of actives [(Ha/Ht)×100]	70.67
6	% Ratio of actives [(Ha/A)×100]	80.30
7	Enrichment factor (E) [(Ha×D)/(Ht×A)]	38.61
8	False negatives [A-Ha]	13
9	False positives [Ht-Ha]	22
10	Goodness of hit score^a^	0.73

^a ^[(Ha/4HtA)(3AtHt)) × (1-((Ht-Ha)/(D-A))]; GH Score of 0.7-0.8 indicates a very good model.

### Database screening

**Figure 3:**
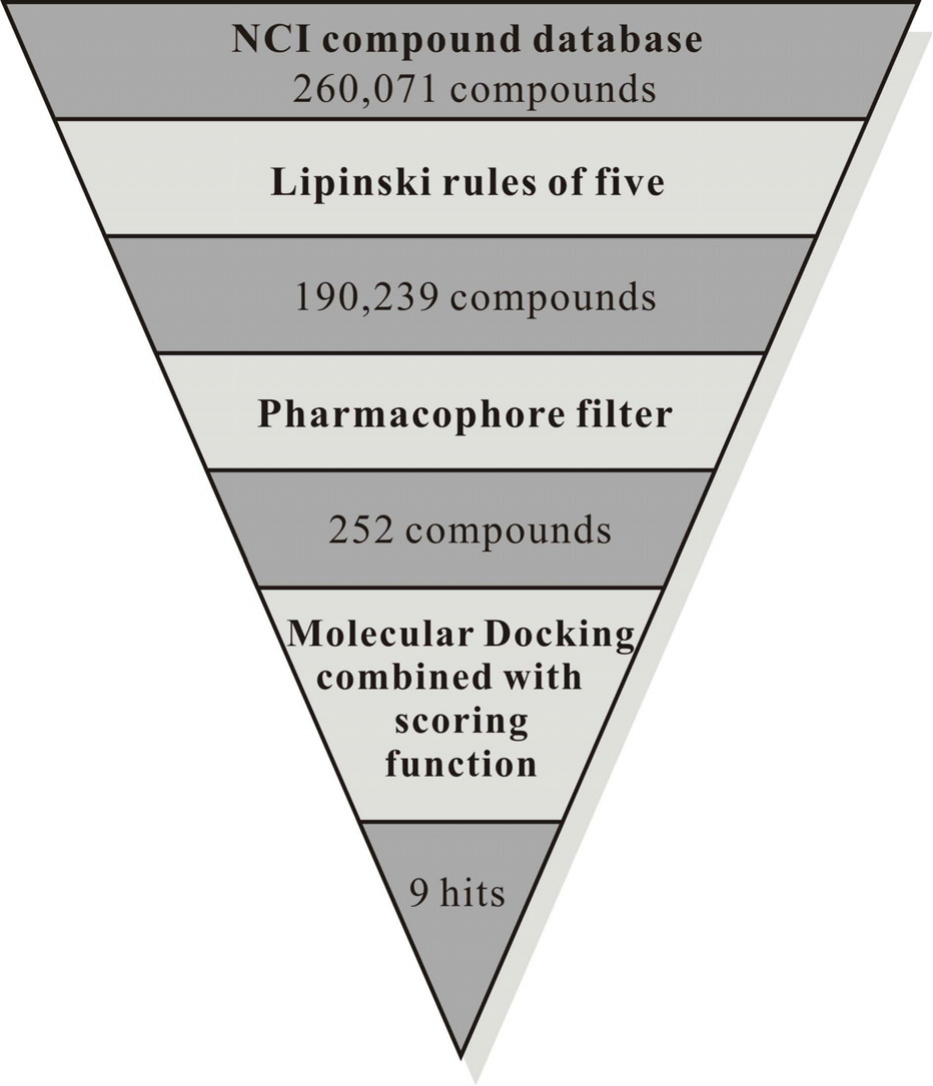
**Schematic representation of virtual screening protocol implemented in the identification of AChE inhibitors**.

One proficient approach to drug discovery is virtual screening of molecule libraries [52]. For conducting virtual screening, we used NCI database containing 260,071 compounds (accessed July 17, 2010). These compounds were first screened for drug like properties using Lipinski rule of 5 as filter [49]. The remaining 190,239 compounds that passed the screening were overlaid with the best 3D pharmacophore model (Hypo1) by using the 'Best Fit' selection. The top 252 hits with the highest fit values were subsequently analyzed for binding patterns using docking methods. The flowchart in Figure 3 is a schematic representation of the sequential virtual screening process with the number of hits reduced for each screening step.

### Molecular docking studies of AChE

**Figure 4:**
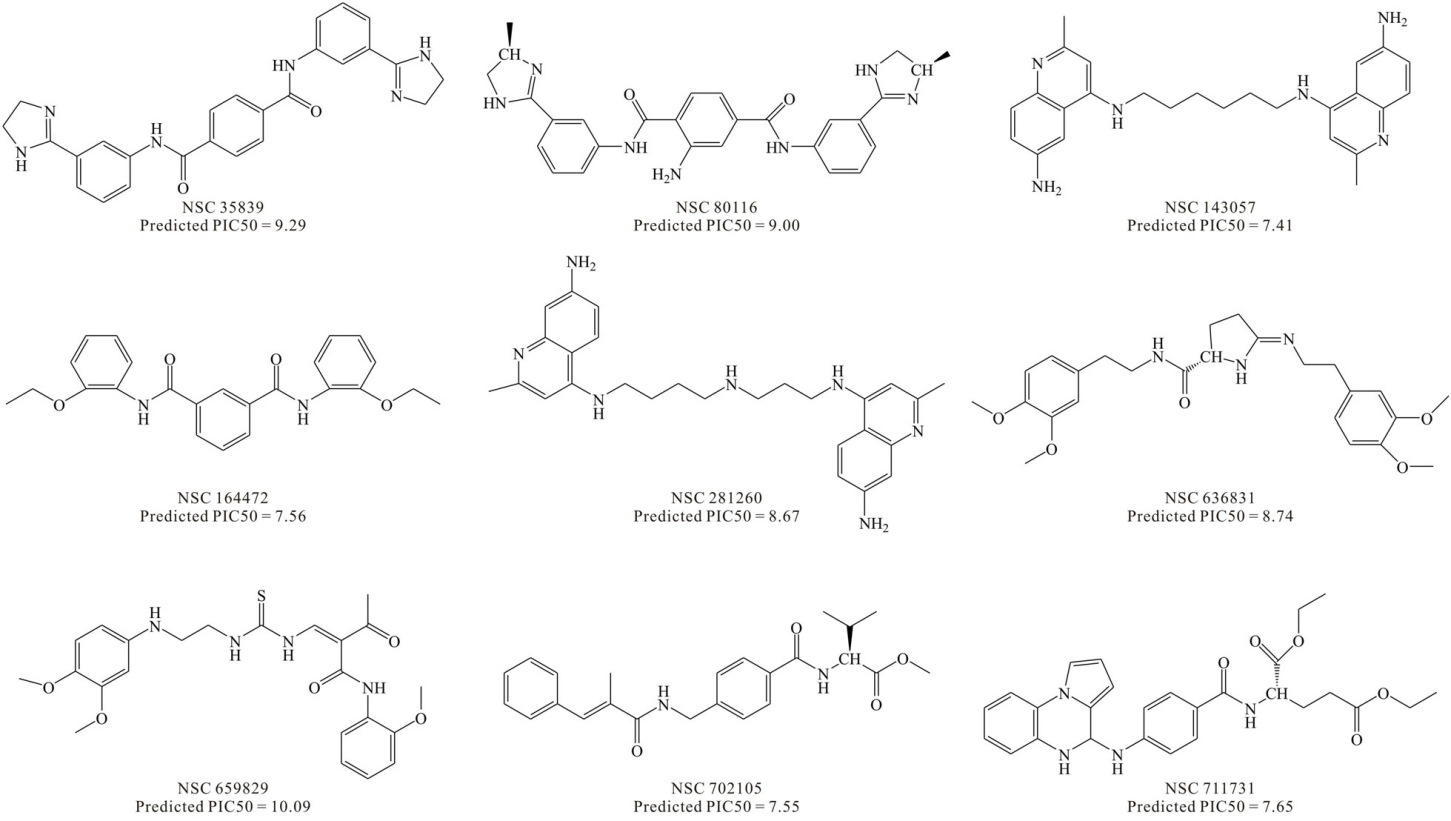
**Lead molecules retrieved from the NCI database as potent AChE inhibitors**. The predicted IC50 values are based on the consensus scoring function.

Docking simulation of AChE (PDB Code: 1B41) [50] and ligands was performed using the LibDock program. The binding modes for the 252 compounds identified by virtual screening were ranked according to the information obtained by different scoring constraints. The 154 highest scoring compounds were selected from a total of 252 compounds for further evaluation. After visual inspection, the most favorable compounds with the best binding modes (exact matching of π-π overlap with residue W86 or π-π overlap with residue W286) and structural diversity were selected. Based on the knowledge of the existing AChE inhibitors and the active site requirements, we selected 9 compounds from the 252 highest scoring structures for subsequent bioactivity prediction and consensus scoring function assay. Information on the molecular docking experiments and the consensus scoring function were taken from a previous study. The 9 hits with the highest binding affinities were ultimately selected after careful observations, analyses and comparisons. The structures of these best hits from the final screening are reported in Figure 4. The highest pose scores extracted from the eleven default scoring methods and the predicted pIC50 values calculated by the consensus scoring function developed in this study for all of the 9 best hits are summarized in Table 3. Among the hits found were some novel structures. The diversity of the hits demonstrated that the pharmacophore model was able to retrieve hits with similar features to the existing AChE inhibitors as well as novel scaffolds.

**Table 3:** The highest pose scores for the most potent AChE inhibitors from the NCI database.

Name		(-PLP1)	(-PLP2)	(-PMF)	(-PMF04)	Jain	LibDockScore	LigScore1	LigScore2	Ludi1	Ludi2	Ludi3	PIC50
NSC	35839	128.01	127.24	259.45	188.38	5.78	152.48	3.65	4.53	808	624	1,333	9.29
NSC	80116	131.87	134.17	270.73	193.51	5.78	154.91	2.63	3.75	782	623	1,374	9.00
NSC	143057	141.36	138.40	215.12	163.47	6.01	162.90	3.79	4.49	691	572	1,069	7.41
NSC	164472	114.29	125.19	202.62	142.87	4.11	129.24	4.20	5.67	761	633	996	7.56
NSC	281260	134.57	139.96	207.72	149.91	8.11	169.53	0.68	-0.58	674	588	1,138	8.67
NSC	636831	128.45	125.65	231.45	166.82	5.73	161.51	0.75	0.13	693	592	984	8.74
NSC	659829	139.17	142.52	207.47	158.28	2.75	143.76	4.30	3.91	826	640	734	10.09
NSC	702105	115.87	114.15	222.21	142.65	4.30	142.34	2.38	2.43	690	549	808	7.55
NSC	711731	131.92	132.18	189.08	130.37	8.01	151.50	1.32	-0.82	619	500	840	7.65

The pose scores are extracted from the eleven default scoring methods with the predicted pIC50 values calculated from the consensus scoring function developed in this study.

## Discussion

In this work, we first generated a qualitative pharmacophore model to effectively map the critical chemical features for AChE inhibitors. The resulting binding hypotheses were automatically ranked based on their "total cost" values, which is the sum of the three costs: error cost, weight cost and configuration cost. As the root mean square difference between the estimated and measured biological activities of the training set molecules increases, so does the error cost. Error cost provides the highest contribution to the total cost [35-38]. HypoGen also calculates the cost of the null hypothesis, with the assumption that there is no relationship between the estimated and measured biological activities. The residual cost (Table 1) is the difference between the cost of null hypothesis and the total cost. The larger the difference between the cost of the null hypothesis and total cost, the greater the likelihood that the correlation between the fit values and actual activities is not a random occurrence [35-38]. The 62 training set molecules were then mapped onto Hypo1 resulting in a correlation coefficient of 0.851, which indicates a good correlation between the actual activities and estimated fit values (Figure 3).

The best pharmacophore model, Hypo1, consists of five features: one hydrogen bond donor and four hydrophobic features. The best quantitative pharmacophore model was further validated by Fischer's randomization test, test set prediction, and Güner-Henry (GH) scoring method. Results of Fischer's randomization test confirmed that the generated hypotheses from the training set are reasonable and that the Hypo1 pharmacophore model has been correctly established. The results obtained by the test set method show good correlation between the experimental activity and the estimated fit values (correlation coefficient of R^2 ^= 0.830) indicating that the pharmacophore model predicted molecular properties well. The results of GH scoring method show that the model is able to identify the active AChE compounds from the database.

**Figure 5:**
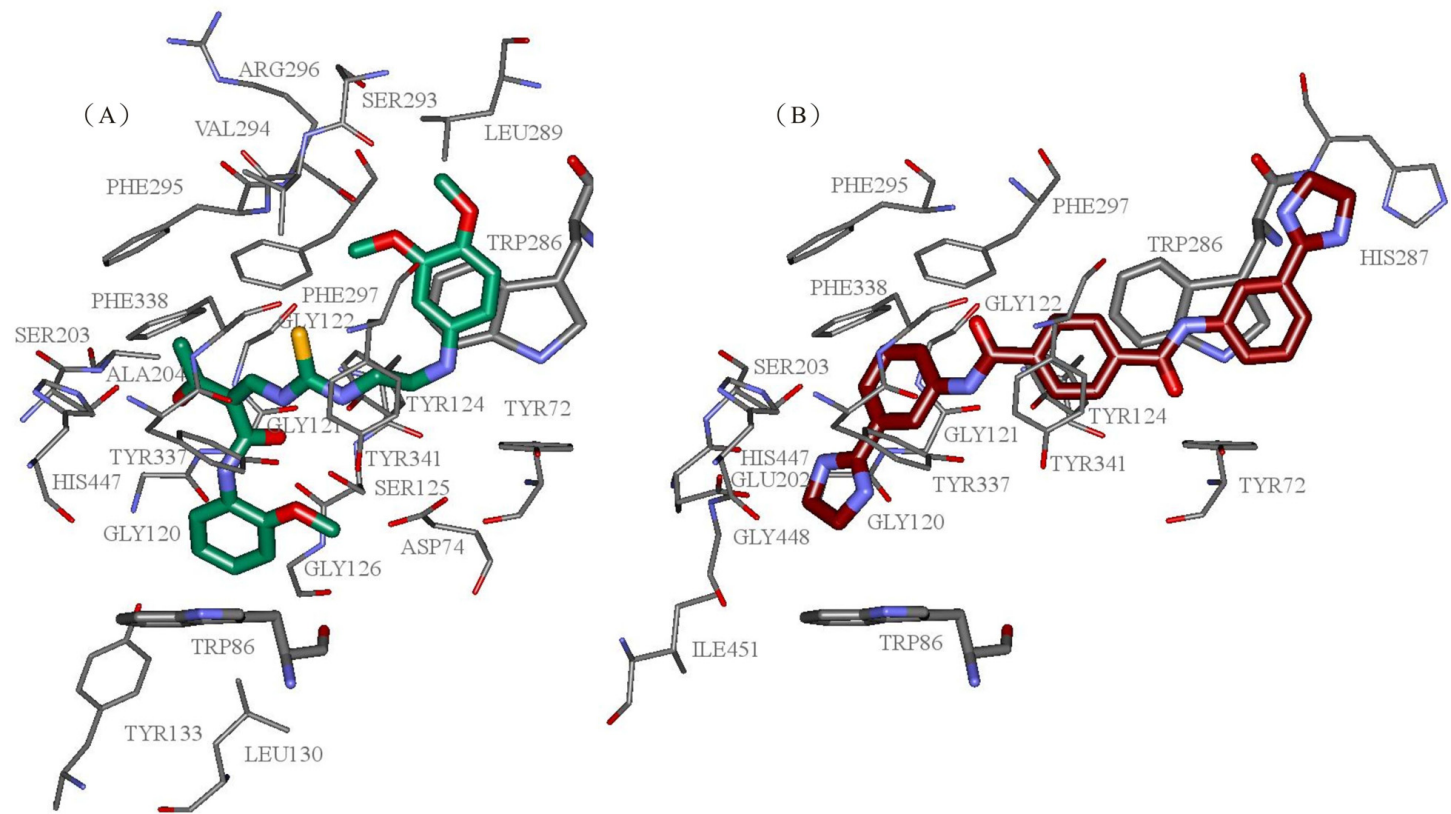
**The binding structures of compounds (A) NSC659829 and (B) NSC35839 after docking into the catalytic gorge of huAChE**. Residues at a distance of less than 4 Å from the compounds are represented as 0.1Å atom-colored sticks. W86 and W286 are displayed as 0.2Å atom-colored sticks. The compounds are shown as 0.3Å stick models (carbon atom in green for (A) and maroon for (B), oxygen atom in red, nitrogen atom in blue, and sulfur atom in yellow).

**Figure 6:**
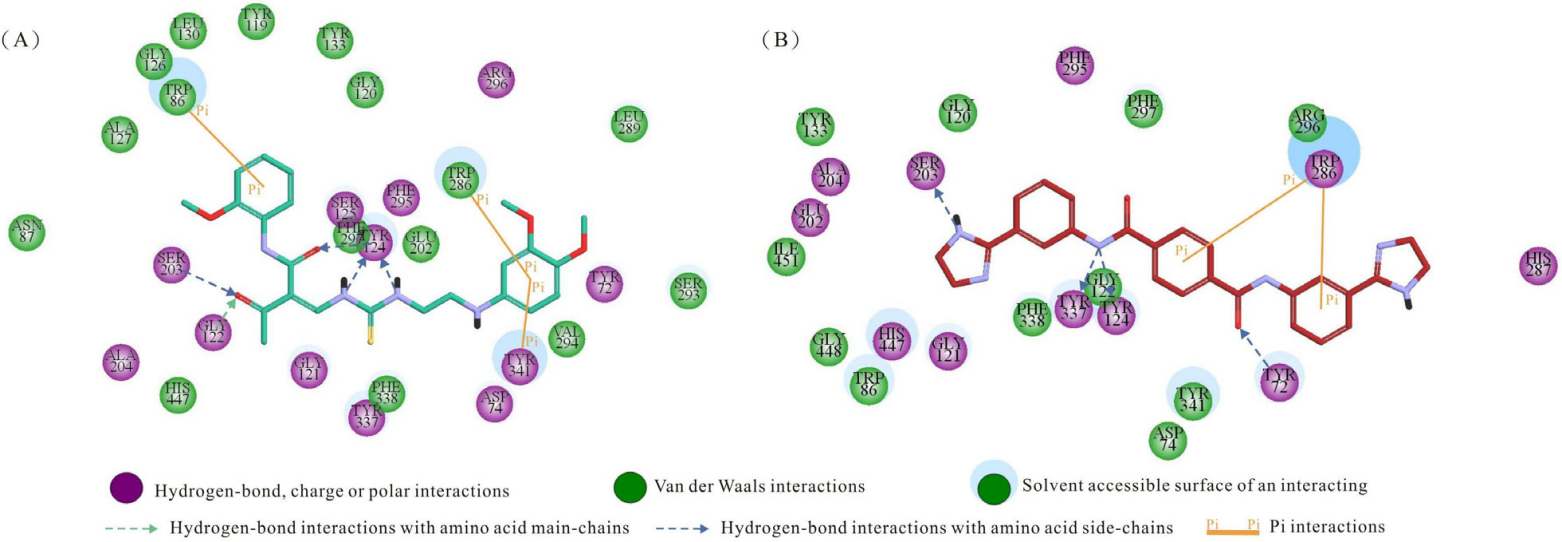
**Schematic presentations of the putative huAChE binding modes with compounds (A) NSC659829 and (B) NSC35839**. Residues involved in hydrogen-bonding, charge or polar interactions are represented by magenta-colored circles. Residues involved in van der Waals interactions are represented by green circles. The solvent accessible surface of an atom is represented by a blue halo around the atom. The diameter of the circle is proportional to the solvent accessible surface. Hydrogen-bond interactions with amino acid side chains are represented by a blue dashed line with an arrow head directed toward the electron donor. π-π Interactions are represented by an orange line with symbols indicating the interaction.

Combining the best pharmacophore model, docking, and finally consensus scoring function activity prediction, we were able to perform virtual screening on a dataset of compounds to identify potential AChE inhibitors and to examine important interactions responsible for binding to AChE. The interactions of the best two compounds (NSC659829 and NSC35839) with the active site of huAChE protein are shown in Figure 5. Figure 6 maps out the interactions between the catalytic gorge of huAChE and the corresponding AChEIs presented in Figure 5. The structure activity relationships of the best hit, NSC659829, against huAChE observed via docking interactions showed that the oxygen and nitrogen functionalities have strong hydrogen bond interactions with S203, G122 and Y124 amino acids present in the active site of huAChE and thus these groups are essential for activity. In the active site, the benzyl rings form a π-π interaction with the indole ring of W86; while in the peripheral site (PS), the benzyl ring forms another π-π interaction with the indole ring of W286.

Docking studies of the NSC35839 compound with huAChE revealed that the oxygen and nitrogen functionalities are making hydrogen interactions with the active site containing Y72, Y124, Y203 and Y337 amino acids. In the PAS, the benzyl ring forms another π-π interaction with the indole ring of W286. Despite the lack of π-π interaction with W86, other interactions were found to play important roles. Hydrogen bonds might be one reason for the enhanced activity of nitro substituted compounds. The proposed interactions of these compounds with W286 in huAChE suggest a possibility to interfere with amyloid fibrillogenesis in addition to inhibiting the catalytic function of the enzyme. The interactions found after docking include π-π stacking contacts with residues in the anionic substrate binding site (Trp86, Phe331, and Tyr334) and the PAS (Trp286). Hydrogen bonding to amino acids is also found at the bottom of the gorge.

The combination of these interactions in other inhibitors (e.g., donepezil, galanthamine) is already found in the AChE crystal complex structure and therefore the docking results also show similarities that are meaningful for the test compounds. In addition, although all compounds are able to bind the active side of the gorge, not all of them are able to interact with all the important residues previously identified at the binding sites. Ligand size may be one reason for some of the activities being low. McCammon and coworkers have previously mentioned this problem with their molecular dynamics studies [53].

In conclusion, the previously mentioned π-π interactions, hydrogen bonds, and strong hydrophobic interactions formed between the inhibitors and the nearby huAChE side chains serve dual roles: 1) to inhibit the catalytic activity of AChE by competing with Ach binding site and 2) to prevent amyloid fibrillogenesis by blocking the Aβ recognition zone at the peripheral site. In light of the pharmacophore model developed in this study and the knowledge gained from the observations of the interactions between huAChE and potential inhibitors, it can be seen that the combination of pharmacophore, molecular docking, and virtual screening efforts is successful for discovering more effective inhibitory compounds that can have a great impact for future experimental studies in diseases associated AChE inhibition.

## Conclusions

The work presented in this study shows that a set of compounds along with their activities ranging over several orders can be used to generate a good pharmacophore model, which in turn can be utilized to successfully predict the activity of a wide variety of chemical scaffolds. This model can then be used as a 3D query in database searches to determine compounds with various structures that can be effective as potent inhibitors and to assess how well newly designed compounds map onto the pharmacophore prior to undertaking any further research including synthesis.

Biological evaluation and optimization in designing or identifying compounds as potential inhibitors of AChE were made possible by the our pharmacophore study that showed the best model of AChE inhibitors were made up of one hydrogen bond donor and four hydrophobic features. The most active molecule in the training set fits the pharmacophore model perfectly with the highest scores. The pharmacophore model was further used to screen potential compounds from the NCI database followed by virtual screening that produced some number of false positives and false negatives. Then we used molecular docking and consensus scoring methods, as added tools for virtual screening to minimize these errors. Through our docking study, the important interactions between the potent inhibitors and the active site residues were determined. Using a combination of pharmacophore modeling, virtual screening, and molecular docking, we successfully identified putative novel AChE inhibitors, which can be further evaluated by *in vitro *and *in vivo *biological tests.

## Author details

Both SL and JW are graduate students in the Graduate Institute of Biotechnology of National Taipei University of Technology under HLL's instruction. HLL is a distinguished professor in the Graduate Institute of Biotechnology of National Taipei University of Technology. JZ is a postdoc fellow in the Chemical Analysis Division of Institute of Nuclear Energy Research under KL's instruction. KL works as a research fellow in the Chemical Analysis Division of Institute of Nuclear Energy Research. CC is a research fellow in the Department of Medical Research of Mackay Memorial Hospital. HYL, WT, and YH are professors from National Taiwan University, National Taipei University of Technology, and Taipei Medical University, respectively.

## Competing interests

The authors declare that they have no competing interests.

## Authors' contributions

SL carried out the entire simulation work presented in this study. JW helped to compose the manuscript. HLL is the corresponding author for this article and made substantial contributions to conception and design of the experiments. JZ participated in the design of the study and performed the statistical analysis. KL, CC, HYL, WT, and YH provide valuable discussion to this work and helped to draft the manuscript. All authors have read and approved the final manuscript.

## Supplementary Material

Additional file 1 **Table S1: The structures of AChE inhibitors utilized in the modeling**. Training set of AChE inhibitors considered for this study, including chemical structures, experimental IC50 and pIC50 values. Click here for file

Additional file 2 **Table S2: The structures of AChE inhibitors utilized in the modeling**. Test set of AChE inhibitors considered for this study, including chemical structures, experimental IC50 and pIC50 values. Click here for file
